# In Vitro Digestibility, Structural and Functional Properties of *Millettia speciosa* Champ. Seed Protein

**DOI:** 10.3390/biom15121722

**Published:** 2025-12-11

**Authors:** Qing Yang, Shuxian Ding, Qinglong Wang, Li Xu, Xiaoxia Yan, Huan Tang, Langxing Yuan, Xiaoyan Chen, Zhunian Wang, Maoyuan Wang

**Affiliations:** 1 Tropical Crops Genetic Resources Institute, Key Laboratory of Biology and Cultivation of Chinese Medicinal Materials, Ministry of Agriculture and Rural Affairs/Hainan Provincial Engineering Research Center for Tropical Medicinal Plants/Key Laboratory of Tropical Crops Germplasm Resources Genetic Improvement and Innovation of Hainan Province, Chinese Academy of Tropical Agricultural Sciences, Haikou 571101, China; yangqing@catas.cn (Q.Y.); dingsx@catas.cn (S.D.); qlwang1983@catas.cn (Q.W.); xllzy@263.net (L.X.); yanxx@catas.cn (X.Y.); tanghuan@catas.cn (H.T.); yuanlangxing@catas.cn (L.Y.); chenxiaoyan@catas.cn (X.C.); wangzhunian@catas.cn (Z.W.); 2 National Genebank of Tropical Crops, Danzhou 571737, China; 3 Sanya Research Institute, Chinese Academy of Tropical Agricultural Sciences, Sanya 572025, China

**Keywords:** *Millettia speciosa* champ. seed protein, physicochemical characteristics, in vitro digestibility, structural properties, functional properties, health-promoting food

## Abstract

As an underutilized industrial byproduct generated during bioactive compound extraction from *Millettia speciosa* Champ. seeds, the residual protein fraction represents a promising sustainable resource for valorization. *Millettia speciosa* Champ. seed protein (MP) was extracted, and its fundamental physicochemical and functional properties were evaluated for potential applications in the food industry. Structural characterization revealed that MP had a molecular weight distribution with major components at 14.0 kDa and 116.0 kDa, with respective denaturation temperatures of 79.75 °C and 91.77 °C. The main structure of MP included different proportions of intramolecular α-helices and random coils in different pH microenvironments, based on circular dichroism spectroscopy. The MP displayed similar solubility profiles to the soy protein isolate (SP), but with lower solubility at slightly acidic pH, low solubility at pH 5.0, and comparable solubility above pH 8.0. Functional assessments showed that MP possessed emulsifying, foaming, water-binding, and fat-absorption capacities comparable to those of SPI, although the in vitro digestibility was relatively lower. These findings indicate that MP may serve as a safe and nutritious functional ingredient for health-oriented food products.

## 1. Introduction

It is estimated that a rise of the world population to 10 billion in 2050 will be seen, which will drive a corresponding increasing demand for food, especially protein [1]. To meet the increasing protein consumption and avoid food insecurity, recognisable and reliable protein sources are attracting increasing interest [2]. Moreover, special attention is being paid to plant protein sources owing to concerns over the safety and environmental friendliness of animal-based protein [3]. In particular, plant protein levels in human diets are increasing rapidly due to nutritional and functional value, as well as economic value.

There are many abundant and renewable proteins in nature that could help to meet to this increasing demand, including those from legumes, rhizomes, wheat, and nuts and seeds. *Millettia speciosa*, a traditional medicinal and edible plant widely distributed in southern China (e.g., Guangxi, Guangdong, and Hainan), possesses diverse pharmacological properties, including immunomodulatory, anti-fatigue, antioxidant, and hepatoprotective activities [4,5,6]. Consequently, it has emerged as a cultivated crop with an annual production exceeding 52,000 tons [7]. Current research predominantly focuses on its cultivation and nutritional components, notably starch, polyphenols, and polysaccharides, while significantly less attention has been paid to its protein constituents [6,7,8,9,10,11]. However, while the starch components of *Millettia speciosa* seeds have been characterized in prior studies [7], investigations into their protein constituents, particularly regarding structural characteristics, remain notably limited. Therefore, elucidating the structural properties of these proteins is crucial for the comprehensive development and utilization of this underutilized byproduct.

This study comprehensively investigated the structural, physicochemical, and functional properties of *M. speciosa* seed protein (MP), including its molecular weight distribution, conformational characteristics, solubility, amino acid composition, emulsifying capacity, foaming capacity, water-binding capacity, and oil-absorption capacity, to evaluate its potential as a sustainable, functionally versatile, and nutritionally enriched ingredient for innovative health-focused food formulations.

## 2. Materials and Methods

### 2.1. Materials

Seeds of *M. speciosa* were collected from the germplasm repository of tropical medicinal plants in October 2021 (Ministry of Agriculture, Danzhou, China). After washing, draining, drying, and grinding, they passed through a 60-mesh screen and were stored at −18 °C. Soy protein (SP; purity 90%) was prepared from defatted soybean meal (Shandong Yuwang Ecological Food Industry, Dezhou, China) according to the classical approaches described by Chen et al. [12]. The study employed pepsin (250 units/mg), pancreatin (4 × UPS specifications), and 8-anilino-1-naphthalene sulfonic acid (ANS), all obtained from Aladdin Biochemical Technology Co., Ltd. (Shanghai, China). Reagent-grade chemicals were consistently used throughout all experimental work.

### 2.2. Protein Preparation

MP was extracted using the alkaline-soluble acid precipitation approach following the method described by Wang et al. [13]. Briefly, defatted *M. speciosa* flour (previously Soxhlet-extracted with hexane) was dispersed in distilled water at a ratio of 1:12 (*w*/*v*) under alkaline conditions (pH 8.5, adjusted with 1.0 M NaOH) and stirred continuously for 4 h at room temperature. After centrifugation (5000× *g*, 30 min, 4 °C), the supernatant was collected and acidified to pH 5.0 using 1.0 M HCl to precipitate the protein. The precipitate was recovered by further centrifugation (5000× *g*, 15 min, 4 °C), washed with ultrapure water, neutralized to pH 7.0, and finally freeze-dried. The resulting protein powder was stored at 4 °C until further use.

### 2.3. Chemical and Proximate Composition Analysis

According to Kjeldahl’s method, protein content was determined. The protein moisture, ash and lipid levels were determined using standard AOAC (2011) methods [14] to be 925.10, 920.85 and 923.03, respectively.

### 2.4. Hydrodynamic Radius (Rh) and ζ-Potential

The protein Rh value was analysed using dynamic light scattering (DLS) [15]. Protein samples (1.0 mg/mL) were filtered through Millipore 0.45 μm filters (Millipore, Shanghai, China). A Master Zetasizer Nano ZS90 particle electrophoresis instrument from Malvern Instruments Ltd., Malvern, UK, was used to perform the DLS measurements. The *ζ*-potential of protein samples was measured via dynamic light scattering (DLS) at 25 °C using a Zetasizer Nano ZS90 (Malvern Panalytical, Malvern, UK).

### 2.5. Circular Dichroism (CD)

The secondary structure of the protein was analyzed by circular dichroism (CD) spectroscopy on a J-810 spectropolarimeter (Jasco Corporation, Tokyo, Japan). Samples (0.5 mg/mL in 10 mM PBS) were adjusted to pH 3.0, 5.0, 7.0, and 9.0 and analyzed using a quartz cuvette with a 0.1 cm path length. Far-UV CD spectra were recorded from 190 to 350 nm at a scanning speed of 20 nm/min with a data interval of 0.2 nm. Secondary structure prediction utilized the online CD Pro server (Fort Collins, CO, USA)

### 2.6. SDS-PAGE Analysis

SDS-PAGE analysis was performed following the protocol of Wang et al. with modifications [15]. Protein samples were prepared in urea buffer (pH 6.8) and subjected to electrophoresis under non-reducing conditions (without *β*-mercaptoethanol) using a 4–20% gradient polyacrylamide gel and a Mini PROTEAN Tetra Cell device (Bio-Rad Laboratories, Hercules, CA, USA) at 100 V constant voltage. Coomassie Blue R-250 staining (Sigma-Aldrich, Shanghai, China) was applied to the loaded gels after electrophoresis and then destaining was applied for 4 h.

### 2.7. Determination of Protein Solubility

Protein solubility at different pH levels was determined according to Jin et al. [16]. Briefly, protein samples (1.0 g) were suspended in 50 mL of deionized water, and the pH was adjusted from 1.0 to 12.0 using 0.5 M HCl or NaOH. The suspensions were stirred at 120 rpm for 2 h at room temperature and then centrifuged at 3000× *g* for 20 min. The soluble protein content in the supernatant was measured using the Lowry method, with BSA as the standard. Solubility was calculated as the percentage of soluble protein relative to the total protein content.

### 2.8. Measurement of Surface Hydrophobicity

The surface hydrophobicity (*H*_0_) of MP was determined using 1-anilino-8-naphthalene sulfonic acid (ANS) as a fluorescent probe, following a procedure adapted from Perović et al. [17]. Fluorescence intensity was recorded at excitation/emission wavelengths of 390/470 nm using a Hitachi F-7000 spectrophotometer (Tokyo, Japan). H_0_ was calculated as the initial slope of the linear regression (R^2^ = 0.95) of fluorescence intensity versus protein concentration.

### 2.9. Thermal Properties Analysis

PE DSC 4000 differential scanning calorimeter (DSC) instrument (Perkin Elmer, Waltham, MA, USA) was used to measure the thermal properties of MP. Protein sample (Approximately 4.0 mg) was precisely weighed into an aluminium liquid pan and 20 μL of deionized water (pH 7.0) was added. Following thermal equilibration at 25 °C for 2 h, differential scanning calorimetry was conducted with a heating program from 20 °C to 120 °C at 5 °C/min. The peak and denaturation temperature (Td) of protein and the enthalpy of denaturation (∆H), evaluated from peak areas and results expressed per weight (g) of protein, were calculated from the thermograms using Pyris 10.0 software (Perkin Elmer).

### 2.10. Amino Acid Analysis

Amino acid was analyzed after hydrolyzing protein samples (20 mg) in 6.0 M HCl at 110 °C for 24 h under vacuum-sealed conditions. The hydrolysates were then assessed utilizing a Hitachi L-8800 automatic amino acid analyzer, with results reported as grams of amino acid per 100 g of protein.

### 2.11. Water- and Oil-Holding Capacities

Water- and oil-holding capacities (WHC and OHC) were measured according to a centrifugation-based method described by Wang et al. [13]. In brief, 2.0 g of MP was distilled in 20 g of water or soybean oil, vortexed for 20 s, and allowed to stand at room temperature for 1 h. The mixtures were then centrifuged at 1500× *g* for 30 min. After decanting the unbound liquid, the weight of the retained water or oil was measured. WHC and OHC were expressed as grams of water or oil held per gram of protein.

### 2.12. Emulsifying and Foaming Properties

#### 2.12.1. Emulsifying Capacity and Stability

The emulsifying activity index (EAI) and emulsion stability index (ESI) were evaluated using a turbidimetric approach following Zou et al. [18]. A mixture was prepared by combining 4 mL of soybean oil with 16 mL of protein solution (10 mg/mL in pH 7.0 phosphate-buffered saline, PBS), and then homogenized at 10,000 rpm for 3 min with an XHF-D high-speed homogenizer (Ningbo Xinzhi Inc., Ningbo, China) to form an emulsion. Aliquots (25 μL) of the emulsion were taken immediately and 30 min after homogenization, and each was diluted with 5 mL of SDS solution (1 mg/mL). Absorbance was measured at 500 nm using a UV-1200 spectrophotometer (Mapada Inc., Shanghai, China). EAI and ESI was calculated according to Equation (1) and Equation (2), respectively:

(1) $${\mathrm{EAI}\;(m}^{2}/g)\;=\;[2\times 2.303\times A_{0}\times DF/I\times \Phi \times C]$$

(2) $${\mathrm{ESI}\;(\min)=[A}_{0}{/(A}_{0}-A_{30})]\times \Delta T$$

where A_0_ and A_30_ are the absorbance (500 nm) at 0 min and 30 min, respectively; I is the optical path (0.01 m); C is the initial protein concentration (g/mL); DF is the dilution factor (200); and Φ is the oil fraction in emulsion (0.20).

#### 2.12.2. Foaming Capacity and Stability

Foaming capacity (FC) and foam stability (FS) were determined according to Kaushik et al. [19]. Fifteen millilitres of protein solution (10 mg/mL) were placed in a measuring cylinder and stirred for 3 min by an XHF-D high-speed homogenizer (Ningbo Xinzhi Inc., Ningbo, China) at 8000 rpm. FC and FS were calculated using Equation (3) and Equation (4), respectively:

(3) $${\mathrm{Foaming}\;\mathrm{capacity}\;(\mathrm{FC},\;\%)=[(V}_{2}-V_{1}{)/V}_{1}]\times 100$$

(4) $${\mathrm{Foam}\;\mathrm{stability}\;(\mathrm{FS},\;\%)=[(V}_{3}-V_{2}{)/V}_{2}]\times 100$$

where V_3_ is the foam volume recorded at 30 min of storage; and V_1_ and V_2_ are the volume of the protein solution before and after wrap gas, respectively.

### 2.13. In Vitro Simulated Gastrointestinal Digestion

In vitro simulated gastrointestinal digestion was performed following a modified protocol of Yu et al. [20]. To start the reaction, 0.5 g of protein sample was added to 20 mL simulated gastric fluid (0.1 mol/L KCl-HCl, pH 2.0) containing 10 mg pepsin) and incubated at 37 °C for 60 min. Thereafter, the mixture was immediately neutralised with 1 mol/L NaOH, and 10 mL of simulated intestinal fluid (20 mg pancreatin in 10 mmol/L phosphate buffer, pH 7.2) was added and incubated at 37 °C for another 120 min with continuous shaking. Next, digestion was terminated in a boiling water bath for 10 min and samples were centrifuged at 8000× *g* for 20 min to precipitate undigested protein. The extent of nitrogen release was computed based on the following formula:

% N Release = (N_0_ − N)/N_0_ × 100% (5)

where N_0_ (mg) represents the TCA-insoluble nitrogen content in the original protein sample; and N (mg) denotes the TCA-insoluble nitrogen remaining after enzymatic digestion.

### 2.14. Statistical Analysis

All analyses were performed in triplicate to ensure reproducibility, with results expressed as mean ± standard deviation (SD). Statistical analyses were conducted using SPSS version 24.0 (SPSS Inc., Chicago, IL, USA). Statistical significance was determined by one-way analysis of variance (ANOVA), followed by Duncan’s multiple range test for post hoc comparison of means.

## 3. Results and Discussion

### 3.1. Proximate Composition

The protein content in *M. speciosa* seeds (MS) was found to be 29.32%, with a moisture content of 5.98% and 16.90% lipids (Table 1). MS exhibit a superior protein content compared to other commonly utilized plant protein sources, including yellow peas (21.1%), adzuki beans (20.5%), and chickpeas (12.1%) [21]. Notably, the protein content of MS is comparable to that of *Moringa oleifera* seeds (27.4%), which are recognized as a prominent non-traditional plant protein source [22]. This makes it an ideal raw material for protein applications. After defatting, the fat content was decreased to 6.55% while protein content increased slightly to 34.50%. Subsequent precipitation significantly enhanced protein content to 80.28% (*p* < 0.05). This lower protein content than others (e.g., 90% for SP after precipitation) may be due to non-protein components such as dietary fiber [12]. MP had slightly higher protein (80.28%) and carbohydrate (12.66%) contents, with lower lipid (0.58%) and ash (2.37%) contents. This indicated that the high protein content was due to the removal of some non-protein components during fractionation. These results suggested that it was feasible to reclaim protein from MS, which could prove beneficial to the industrial processing of this edible plant protein from a natural raw material.

### 3.2. Physicochemical Properties of MP

#### 3.2.1. SDS-PAGE Analyses

**Figure 1:**
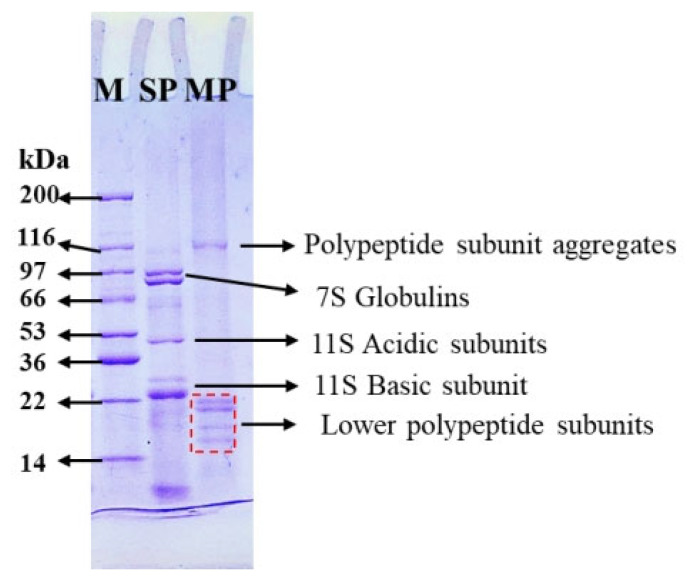
SDS-PAGE profiles of MP compared to SP.

The protein profile of MP was determined by SDS-PAGE under nonreducing conditions. As illustrated in Figure 1, the MP protein is complex, containing five different bands ranging in molecular weight from 14 to 116 kDa. MP yielded fewer bands with higher and lower molecular weights than SP, which corresponds to small polypeptide subunits and their aggregates, respectively. These differences may reflect their diverse categories, and a relatively low level of their corresponding protein fractions was observed previously in *Millettia speciosa* seeds [20]. Additionally, bands at 14–22 kDa were obvious in the SDS-PAGE profile, indicating that MP with numerous small protein subunits may contribute to its functional activities, including emulsifying, and surface and digestive properties [23,24].

#### 3.2.2. Thermal Properties

Protein thermodynamic studies are essential for understanding functional mechanisms, as elevated thermal energy disrupts the stabilizing molecular interactions within proteins, resulting in structural rearrangements and conformational transitions [25]. These protein structural and conformational changes can be characterized using differential scanning calorimetry (DSC). DSC data (Figure S1 and Table 2) demonstrated two characteristic endothermic peaks for MP, reflecting the thermal behavior of individual polypeptide chains and their assembled complexes. The two onset temperatures (*T*_0_) were around 74.87 °C and 84.04 °C, indicating that these structures became less thermally stabile. Peak denaturation (*T*p) values were approximately 79.65 °C and 91.77 °C, demonstrating reduced thermal stability relative to flaxseed protein (105 °C), hemp protein (95 °C) and pumpkin protein (96 °C and 93 °C) [19,26]. MP displayed a ΔH of 3.2 J/g, below the values of 8.25 J/g and 6.26 J/g reported for flaxseed and peanut proteins, respectively [27]. The low ΔH indicates minimal heat absorption per unit mass during the thermal transition [25].

#### 3.2.3. Circular Dichroism

CD spectra of MP at different pH are summarized in Table 3. The spectrum exhibited a positive peak at approximately 190 nm, a zero crossover at 210 nm, and a negative peak around 220 nm. These characteristics confirmed that the secondary structures of MP samples predominantly comprised α-helices and β-sheets [28]. However, MP showed different secondary structure composition in different pH microenvironments (see Table 3 and Figure S2). MP contained greater *β*-sheet content (30.99 ± 0.57%), lower *α*-helix content (17.52 ± 0.54%) and greater random coil content (35.77 ± 0.04%) at pH 5.0, indicating large conformational changes of protein molecules. In contrast, MP displayed a high *α*-helix content at pH 3.0, 7.0 and 9.0. Greater denaturation at pH 5.0 may be attributed to more neutrally charged MP at this pH decreasing electrostatic repulsion between molecules, resulting in larger conformational changes [29]. The differences in secondary structure composition may result in differences in functional properties [28]. Our findings align with earlier research showing that pH changes significantly affect protein structural conformation [30].

#### 3.2.4. Hydrodynamic Radius and Surface Charge (*ζ*-Potential)

**Figure 2:**
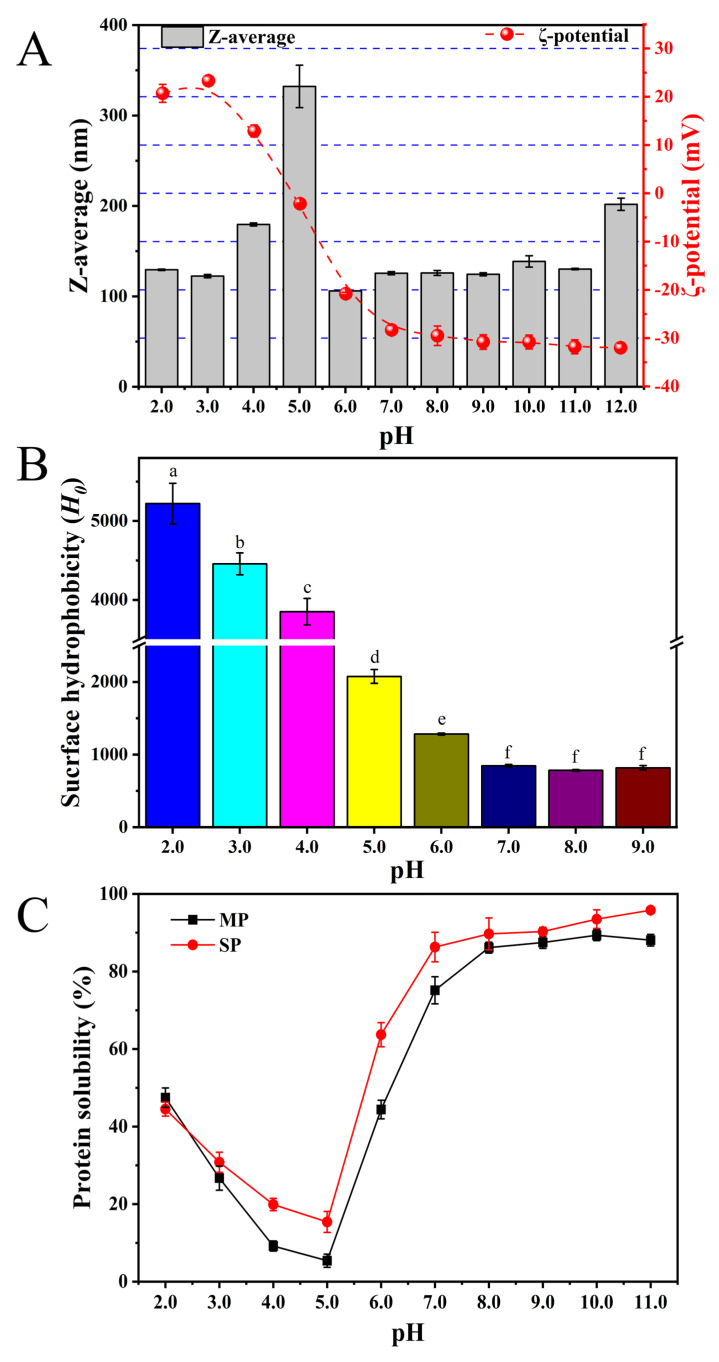
(**A**) The z-average diameter (*D*z) and *ζ*-potential of MP as a function of pH. (**B**) Surface hydrophobicity (*H*_0_) of MP at different pH values. Different superscript letters above the bars indicate significant differences (*p* < 0.05). (**C**) Protein solubility profiles of MP and SP at different pH values. Each value is the mean and standard deviation of triplicate measurements.

The hydrodynamic radius of the protein samples was evaluated across different pH conditions (Figure 2A). At pH 7.0, MP exhibited a hydrodynamic radius of approximately 125.6 ± 1.7 nm, which was significantly larger than that reported for SP [13]. The largest particle size was observed at pH 5.0, likely due to diminished electrostatic repulsion promoting protein aggregation via hydrophobic interactions, hydrogen bonding, and van der Waals forces [31,32]. A similar pH dependence was previously reported for hydrodynamic radius [33]. This reflects the degree of electrostatic stabilization between protein molecules, while surface charge (ζ-potential) is mainly attributed to surface ionisable groups, and this strongly influences functional properties such as solubility, digestibility and emulsifying capacities [34]. As shown in Figure 2A, the electric charge of MP was around zero near pH 5.0, making this the isoelectric point (pI). This finding was consistent with the solubility profile, which showed minimum solubility at pH 5.0 (Figure 2C). The reduced ζ-potential decreased progressively from 20.7 ± 1.9 to −32.0 ± 0.9 mV with increasing pH, indicating surface group ionisation [26]. Moreover, the ζ-potential of MP was highly negative in neutral and alkaline microenvironments, resulting in strong negative repulsion among protein molecules and a tendency to be stable.

#### 3.2.5. Surface Hydrophobicity (*H*_0_)

The *H*_0_ value is an important predictor of protein functional features such as solubility and emulsifying ability [17]. As illustrated in Figure 2B, the *H*_0_ of MP decreased significantly (*p* < 0.05) as pH increased from 2.0 to 9.0, likely due to conformational changes resulting from protonation of charged amino acid residues on the protein surface [28]. At pH 7.0, the *H*_0_ of MP was notably higher than that reported for SP (746.4) [35]. Hydrophobic interactions play a dominant role at the oil–water interface, where nonpolar groups are essential for emulsion formation. The high *H*_0_ value of MP promoted better molecular organization at the interface, thereby improving its emulsifying capacity owing to enhanced hydrophobic character.

### 3.3. Functional Properties of MP

#### 3.3.1. Protein Solubility

Protein solubility is a critical physicochemical property that directly influences functional characteristics, including emulsification, foaming, and gelation capacity, thereby affecting its applicability in food formulations. As shown in Figure 2C, the solubility of MP in water varied with pH, similar to SP that is used extensively in industry. MP and SP showed similar solubility trends, with nearly U-shaped traces corresponding to a strong pH-dependent solubility profile. Although MP showed lower solubility than SP at various pH values, it had a higher pI (pH 5) than SP (pH 4), which may be attributed to the molecular weight, composition and surface characteristics of constituent amino acids [36].

#### 3.3.2. Amino Acid Analysis and Chemical Score

A comprehensive analysis of amino acid composition is essential for elucidating protein structure, conformation, and functionality. As summarized in Table 4, eighteen naturally occurring amino acids were identified in MP, all basic components of a protein source. Furthermore, MP was found to be rich in phenylalanine and tyrosine (80.62 mg/g), aspartic acid (58.35 mg/g) and glutamic amino acid (169.25 mg/g), consistent with the *H*_0_ and solubility results (Figure 2B,C), reflecting the formation of hydrogen bonds. In terms of nutrition, MP could provide improved nutritional quality to poorly balanced proteins due to its high concentration of these amino acids [36]. Nutritionally, MP demonstrated a well-balanced amino acid profile comparable to those of soy protein (SP) and casein, both recognized as high-quality protein references. It’s essential amino acid content meets or exceeds the FAO/WHO/UNU scoring patterns, with threonine, valine, phenylalanine, and tyrosine all present in ample amounts. Although the EAA/NEAA ratio of MP (59.86%) was slightly below that of SP (67.30%) [36] and casein (63.01%) [37], it remained close to the FAO/WHO recommended threshold (≥60%). These results affirmed that MP represented a nutritionally valuable and promising protein source for food applications.

#### 3.3.3. Water-Holding and Fat Absorption Capacities

Water-holding capacity (WHC) and oil-holding capacity (OHC) are key functional properties that reflect a protein’s ability to retain water and bind oil, respectively, contributing significantly to the texture and sensory attributes of food products. As presented in Table 5, MP demonstrated a WHC of 5.61 g H_2_O/g protein, which is similar to that of SP (5.95 g H_2_O/g protein), suggesting comparable hydration abilities likely associated with its protein and crude fiber content [38]. Furthermore, the OHC of MP (6.24 g oil/g protein) exceeded that of both SP (5.09 g oil/g protein) and cowpea protein (1.10 g oil/g protein) [38]. This enhanced oil-binding capacity may result from lower solubility and reduced accessibility of polar amino acids in MP (Figure S2, Table 4). When compared to peanut protein [39], MP showed superior WHC and OHC, indicating improved functionality in both water retention and oil absorption.

#### 3.3.4. In Vitro Digestibility

An in vitro simulation of MP digestion was assessed by comparison with SP by measuring TCA-soluble nitrogen release. As shown in Table 5, MP digestibility (73.61 ± 2.51%) was markedly higher than that of buckwheat (43.30–61.2%), wheat (52.7%), sorghum (59.1%), rice (59.4%) and maize (66.6%), and slightly lower than SP (77.86 ± 3.01%) [40]. The observed digestibility differences may arise from heterogeneous susceptibility of protein subunits to pepsin-catalysed hydrolysis [41]. The in vitro digestibility of MP can be considered comparable (*p* > 0.05) with commercial SP. However, the in vitro digestibility of MP (57.18 ± 0.82%) was significantly (*p* < 0.05) lower than that of SP (62.64 ± 1.05%) when pepsin alone was applied. The limited in vitro pepsin digestibility of MP appears to be associated with its lower solubility characteristics (Table 1). However, the overall functional properties demonstrate that MP has significant potential as a functional food ingredient.

### 3.4. Emulsion and Foam Properties

#### 3.4.1. Influence of pH on Emulsification Performance of MP

**Figure 3:**
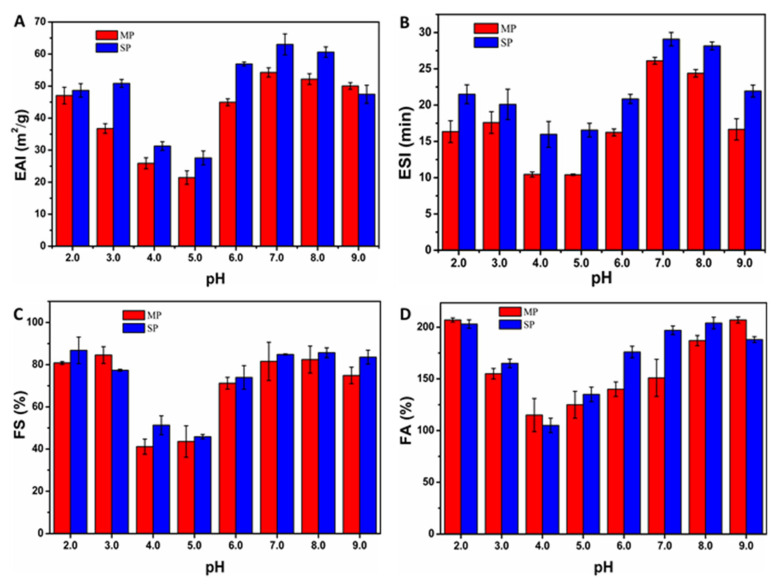
Effect of pH on the emulsifying ability (**A**) and emulsion stability (**B**) of *Millettia speciosa* protein (MP) and soy protein (SP). Effect of pH on the foaming ability (**C**) and foam stability (**D**) of *Millettia speciosa* protein (MP) and soy protein (SP).

The emulsifying properties of MP were assessed by measuring the emulsion activity index (EAI) and emulsion stability index (ESI). As shown in Figure 3A,B, the lowest EAI value (24.43 ± 7.12 m^2^/g) was recorded at pH 5.0, while the highest (60.18 ± 1.69 m^2^/g) occurred at pH 7.0. The minimal EAI near pH 5.0 aligned with the protein’s isoelectric point (pI), where charge neutralization reduces electrostatic repulsion among proteins, impairing emulsification [42]. Under strongly acidic or alkaline conditions, protein unfolding exposed hydrophobic residues normally embedded within the structure, thereby improving emulsion formation and stability. Similarly, ESI values were lowest around pH 4.0–5.0 (Figure 3B), mirroring the EAI trend and correlating with MP’s pI and solubility profile. While emulsifying capacity was pH-dependent, with alkaline conditions conferring greater improvement than acidic conditions, possibly due to changes in the protein’s hydrophilic-lipophilic balance across pH. This pH-dependent behavior was consistent with earlier reports [43]. Weakened repulsive forces near the pI promoted droplet coalescence and reduced emulsion stability. The ESI-pH relationship for MP correlated positively with nitrogen solubility (Figure 3D), supporting the established link between emulsification performance and protein solubility [41].

#### 3.4.2. Influence of pH on Foaming Performance of MP

As shown in Figure 3C,D, the foaming capacity (FC) and foaming stability (FS) of MP varied with pH. No foaming occurred near the isoelectric region (pH 4.0–5.0), likely due to its proximity to the protein’s pI (Figure 2C). Furthermore, improved foaming performance was observed at pH values above and below the pI. FC was strongly associated with protein solubility and amphiphilicity, which facilitated a reduction in surface tension at the air–water interface and improve FS [44]. Consistent with this, foaming was weakest near the pI, where protein solubility was minimal. Enhanced foaming under acidic and alkaline conditions results from an increase in net protein charge, which weakened intramolecular hydrophobic interactions and improves molecular flexibility. These structural changes supported faster protein adsorption at the air–water interface, promoting the formation and stabilization of bubbles [44]. Therefore, MP showed potential for use in aerated foods, given its foaming properties are comparable to those of SP (Figure 3C,D).

## 4. Conclusions

The present study characterized the physicochemical and functional properties of a novel plant protein isolated from *M. speciosa* (MS) seeds to evaluate its potential for food use. SDS-PAGE analysis revealed that MP was composed of subunits ranging from 14.0 to 116.0 kDa, with two thermal denaturation peaks observed at 79.75 °C and 91.77 °C. CD spectroscopy indicated that the secondary structure of MP consisted of varying proportions of α-helices and random coils under different pH conditions. And solubility was lowest near the isoelectric point (pH 5.0). Nutritionally, MP contained all essential amino acids, comparable to soy protein (SP) and casein, and satisfied the WHO/FAO amino acid requirements for both infants and adults. Functionally, MP showed emulsifying, foaming, water-holding, and oil-holding capacities similar to those of SP, although in vitro digestibility was significantly reduced. These findings supported the potential of MP as a viable and functional plant-based protein source for nutraceutical and health-promoting food applications.

## Figures and Tables

**Table 1:** Proximate compositions analysis of *Millettia speciosa* Champ. seed, defatted flour and its protein ^A^.

Sample	Moisture (%)	Protein (%)	Ash (%)	Lipids (%)	Total Carbohydrates (%)
*Millettia speciosa* seeds	5.98 ± 0.28 ^a^	29.32 ± 2.18 ^b^	5.63 ± 0.22 ^a^	16.90 ± 1.98 ^a^	42.17 ± 3.54 ^b^
Defatted flour	6.35 ± 0.12 ^a^	34.50 ± 0.66 ^b^	4.58 ± 0.17 ^b^	6.55 ± 0.41 ^b^	49.02 ± 2.51 ^a^
MP ^B^	4.11 ± 0.30 ^b^	80.28 ± 2.16 ^a^	2.37 ± 0.23 ^c^	0.58 ± 0.04 ^c^	12.66 ± 1.26 ^c^

^A^ All data were based on the dry basis. ^B^ MP is the protein from *Millettia speciosa* seeds. Each value was the mean and standard deviation of triplicate measurements. The letters (^a–c^) indicate significant (*p* < 0.05) difference within the same column.

**Table 2:** DSC Characteristics parameters of MP.

Peak	*T*_o_ (°C)	*T*_p_ (°C)	*T*_e_ (°C)	ΔH (J/g)
Peak 1	74.87 ± 0.62 ^b^	79.65 ± 0.22 ^b^	83.38 ± 0.33 ^b^	0.63 ± 0.03 ^b^
Peak 2	84.04 ± 0.41 ^a^	91.77 ± 0.05 ^a^	100.48 ± 2.09 ^a^	2.58 ± 0.11 ^a^

The letters (^a,b^) indicate significant (*p* < 0.05) difference within the same column. *T*_o_: On-set temperature of denaturation (*T*_o_); *T*_p_: Thermal denaturation temperature peak; *T*_e_: End-set temperature of denaturation; ΔH: Enthalpy changes of the endotherm.

**Table 3:** Secondary structure composition of MP at pH 3.0, 5.0, 7.0 and 9.0.

Secondary Structure	Content (%)			
	pH = 3.0	pH = 5.0	pH = 7.0	pH = 9.0
α-helix	37.15 ± 0.21	17.52 ± 0.54	33.05 ± 0.49	35.20 ± 0.56
β-sheet	15.15 ± 0.10	30.99 ± 0.57	19.60 ± 0.71	17.60 ± 0.55
β-turn	16.90 ± 0.14	16.05 ± 0.05	17.75 ± 0.35	17.55 ± 0.35
Random coil	24.55 ± 0.78	35.77 ± 0.04	26.20 ± 0.14	23.60 ± 0.12

Results are expressed as the mean ± standard deviation (n = 3).

**Table 4:** Comparison of essential amino acid content of MP, soy protein, and casein to FAO/WHO suggested requirements amino acid composition.

Amino Acid	Amino Acid Pattern (mg/g)			WHO/FAO Suggested Requirements ^C^ (mg/g)		
	Protein Samples			Age (Years)		
	MP	SP ^D^	Casein ^E^	1–2	3–10	>18
Asp	58.35	130.2	33			
Thr	33.51	35.7	34	27	25	23
Ser	33.82	64.6	42			
Glu	169.25	208.2	157			
Gly	23.57	37.6	15			
Ala ^A^	30.64	32.3	22			
Cys	2.84	13.1	ND			
Val	44.03	39.3	51	42	40	39
Met + Cys ^A^	25.31	26.2	27	26	24	22
Ile ^A^	43.07	42.0	38	31	31	30
Leu ^A^	52.72	71.1	61	63	61	59
Tyr	48.50	35.4	51			
Phe + Tyr ^A^	80.62	85.6	81	46	41	38
Lys ^A^	32.80	56.8	49	52	48	45
His ^A^	15.04	29.5	15	18	16	15
Arg	28.64	78.1	22			
Pro	25.56	49.8	60			
EAA/NEAA ^B^ (%)	59.86	67.30	63.01			

^A^ Essential amino acids. ^B^ EAA/NEAA = Essential amino acid/Nonessential amino acids. ^C^ WHO/FAO = World Health Organization/Food and Agriculture Organization. ^D^ Data from Han et al. [36]. ^E^ Data from Rubio & Seiquer, I. [37]. ND: Not detected. Values represent the mean of duplicate measurements.

**Table 5:** Water holding, fat absorption capacities and In vitro pepsin (and trypsin) digestibility (%N release) of MP compared to SP.

Sample	WHC (g/g)	OAC (g/g)	In Vitro Digestibility (% *N* Release)	
			Pepsin ^A^	Pepsin + Trypsin ^B^
MP	5.61 ± 0.33 ^a^	6.24 ± 0.21 ^b^	57.18 ± 0.82 ^a^	73.61 ± 2.51 ^a^
SP	5.95 ± 0.21 ^b^	5.09 ± 0.26 ^a^	62.64 ± 1.05 ^b^	77.86 ± 3.01 ^a^

WHC: water holding capacities; OAC: oil absorption capacities; MP: *Millettia speciosa *Champ. Protein; SP: soy protein. ^A^ The % N release of protein after pepsin digestion for 60 min. ^B^ The % N release of protein after pepsin digestion for 60 min and further trypsin digestion for another 120 min. Values are means and standard deviations (*n* = 3). Different superscript characters indicate significant (*p* < 0.05) difference within column.

## Data Availability

The original contributions presented in the study are included in the article/supplementary material, further inquiries can be directed to the corresponding authors.

## Supplementary Materials

The following supporting information can be downloaded at: https://www.mdpi.com/article/10.3390/biom15121722/s1, Figure S1: DSC thermogeams of MP; Figure S2: CD spectra of MP at pH 3.0, 5.0, 7.0 and 9.0.
