# Supplementary material for: In Vitro Digestibility, Structural and Functional Properties of Millettia speciosa Champ. Seed Protein
Source: Biomolecules. 2025 Dec 11;15(12):1722. doi: 10.3390/biom15121722 (PMC12730287; doi:10.3390/biom15121722)
Supplement: Supplementary file 1 [file biomolecules-15-01722-s001.zip › biomolecules-3984211-supplementary.pdf]

Supplementary material

In vitro digestibility, structural and functional properties of *Millettia speciosa* Champ. seed protein

Qing Yang [1, †], Shuxian Ding [1, †], Qinglong Wang [1], Li Xu [1,3], Xiaoxia Yan [1], Huan Tang [1], Langxing Yuan [1], Xiaoyan Chen [1,2], Zhunian Wang [1,2], Maoyuan Wang [1*]

[1] Tropical Crops Genetic Resources Institute, Key Laboratory of Biology and Cultivation of Chinese Medicinal Materials, Ministry of Agriculture and Rural Affairs /Hainan Provincial Engineering Research Center for Tropical medicinal plants/ Key Laboratory of Tropical Crops Germplasm Resources Genetic Improvement and Innovation of Hainan Province, Chinese Academy of Tropical Agricultural Sciences, Haikou 571101, China

[2] Sanya Research Institute, Chinese Academy of Tropical Agricultural Sciences, Hainan Sanya 572025, China

[3] National Genebank of Tropical Crops, Danzhou 571737, China

* Correspondence: wmy81@163.com

† These authors contributed equally to this work.

Figure S1

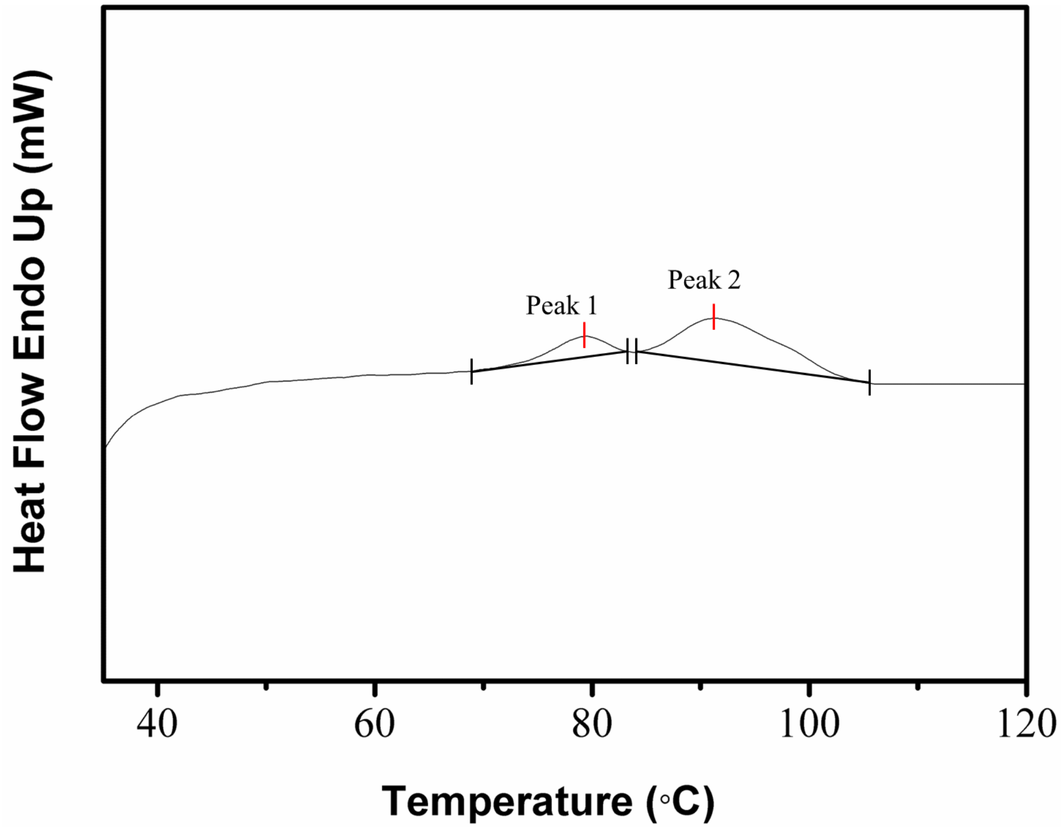

Figure S1 DSC thermogeams of MP.

Figure S2

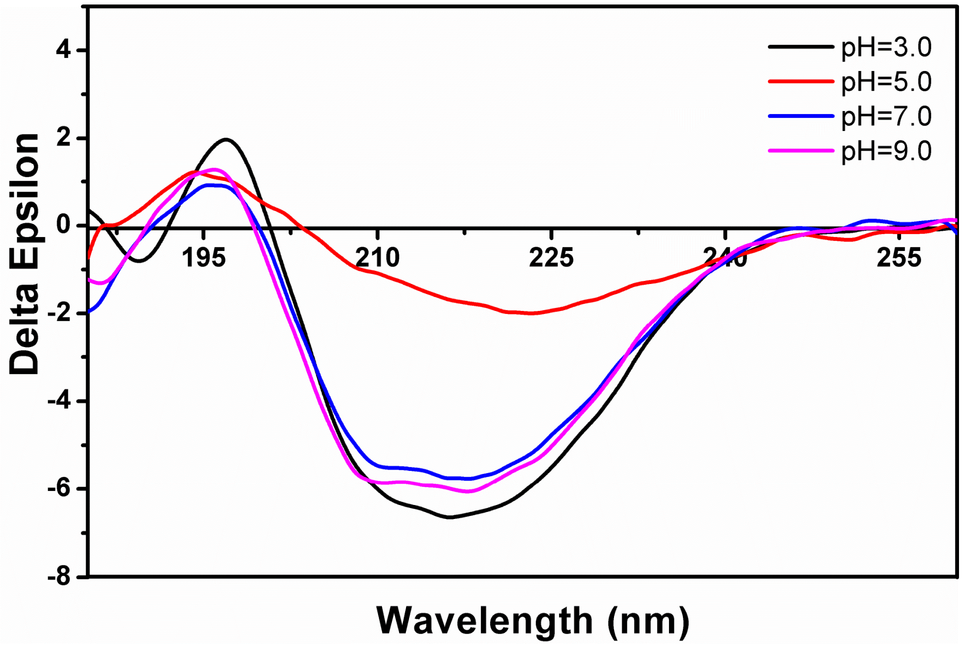

Figure S2 CD spectra of MP at pH 3.0, 5.0. 7,0 and 9.0.
